# Sun Exposure and Protection Practices Among Youths in Canada

**DOI:** 10.1001/jamanetworkopen.2025.51872

**Published:** 2026-01-05

**Authors:** Amina Moustaqim-Barrette, Hiba Elhaj, Meghan Kanou, Elena Netchiporouk, Ivan V. Litvinov

**Affiliations:** 1 Faculty of Medicine and Health Sciences, McGill University, Montréal, Québec, Canada; 2 Division of Experimental Medicine, McGill University Health Centre, Montréal, Québec, Canada; 3 Department of Specialized Medicine (Dermatology), St Mary’s Research Centre, St Mary’s Hospital Centre, Montreal, Quebec, Canada

## Abstract

This survey study analyzes data from the Canadian Community Health Survey to investigate sun protection practices among individuals aged 12 to 19 years.

## Introduction

Most melanoma and nonmelanoma skin cancers result from excessive ultraviolet (UV) exposure, with approximately half of lifetime UV exposure occurring during childhood and adolescence.^1^ Recent mendelian randomization studies support a causal association between childhood sunburn (ie, skin reddening, inflammation, blistering, or peeling) and melanoma and nonmelanoma skin cancers.^2^ The most recent national data on Canadian youth sun protection behaviors come from the National Sun Surveys of 1996 and 2006, which excluded adolescents aged 13 to 15 years.^3^ This study aimed to quantify UV exposure, sun protection practices, sunburn prevalence, and sociodemographic factors for sunburn among Canadian adolescents aged 12 to 19 years. We hypothesized that adolescents would have higher sunburn rates and inconsistent protective behaviors, reflecting reduced parental supervision compared with younger children.

## Methods

This survey study analyzed nationally representative data from the 2015 to 2018 cycles of the Canadian Community Health Survey (CCHS) for adolescents aged 12 to 19 years. Multivariable logistic regression estimated odds of sun exposure, protection, and sunburn outcomes. Full survey methodology and analytic details are available elsewhere^4^ and in the eTable and eMethods in Supplement 1. The study adhered to the AAPOR reporting guidelines. Because it used open-source CCHS data (Statistics Canada and/or Canadian Institute for Health Information), under Canadian law institutional review board ethics review was not required. Results are reported as adjusted odds ratios (ORs) with 95% CIs. Analyses were conducted in R version 4.3.3 (R Project for Statistical Computing). Associations were considered statistically significant if the 95% CI for a given OR did not include 1 (corresponding with a *P* < .05).

## Results

The weighted sample included 7139 Canadian youths, representing 2 276 894 youths (Table). Overall, 40.5% of adolescents reported at least 1 sunburn in the previous year. Most (90.6% of respondents) reported at least 30 minutes of outdoor sun exposure on summer days, while protective measures were inconsistently practiced: 61.5% reported rarely or never applied body sunscreen, 72.8% rarely or never wore hats, and 75.0% rarely or never wore long clothing. Tanning bed use was uncommon (1.7%) and was concentrated among 18- to 19-year-olds.

**Table.** Summary Characteristics of Canadian Community Health Survey Respondents

Characteristics	Respondents, No. (%)							
	Age 12-14 y		Age 15-17 y		Age 18-19 y		Total	
	Unweighted (n = 2859)	Weighted (n = 801 675)	Unweighted (n = 2843)	Weighted (n = 820 072)	Unweighted (n = 1437)	Weighted (n = 655 147)	Unweighted (N = 7139)	Weighted (N = 2 276 894)
Sex								
Male	1450 (50.7)	402 848 (50.3)	1468 (51.6)	429 605 (52.4)	733 (51.0)	342 931 (52.3)	3651 (51.1)	1 175 384 (51.6)
Female	1409 (49.3)	398 828 (49.7)	1375 (48.4)	390 467 (47.6)	704 (49.0)	312 215 (47.7)	3488 (48.9)	1 101 510 (48.4)
Race								
Indigenous	192 (7.0)	37 699 (4.9)	206 (7.4)	45 900 (5.8)	112 (8.0)	30 899 (5.0)	510 (7.4)	114 497 (5.2)
Visible minority^a^	471 (17.1)	239 958 (31.0)	453 (16.4)	231 989 (29.2)	253 (18.1)	180 987 (29.0)	1177 (17.0)	652 934 (29.8)
White	2096 (76.0)	496 054 (64.1)	2111 (76.2)	516 219 (65.0)	1029 (73.8)	411 759 (66.0)	5236 (75.6)	1 424 031 (65.0)
Annual household income, $								
<40 000	415 (14.6)	142 826 (17.9)	456 (16.2)	185 067 (22.7)	362 (25.4)	158 456 (24.3)	1233 (17.4)	486 350 (21.4)
40 000-80 000	746 (26.2)	226 601 (28.3)	723 (25.6)	213 352 (26.1)	364 (25.5)	176 000 (27.0)	1833 (25.8)	615 952 (27.1)
>80 000	1685 (59.2)	430 272 (53.8)	1643 (58.2)	418 509 (51.2)	700 (49.1)	318 577 (48.8)	4028 (56.8)	1 167 357 (51.4)
Time spent in sun								
0 to <30 min	189 (7.0)	60 044 (8.0)	204 (7.5)	68 640 (8.7)	152 (10.9)	74 939 (11.8)	545 (8.0)	203 622 (9.4)
30 min to <2 h	753 (28.0)	228 596 (30.5)	742 (27.2)	225 800 (28.7)	361 (25.9)	179 644 (28.3)	1856 (27.3)	634 040 (29.2)
2 to <4 h	1001 (37.3)	281 169 (37.5)	964 (35.4)	282 256 (35.9)	519 (37.3)	246 307 (38.7)	2484 (36.5)	809 733 (37.3)
4 to 6 h	743 (27.7)	179 505 (24.0)	816 (29.9)	208 986 (26.6)	360 (25.9)	134 752 (21.2)	1919 (28.2)	523 244 (24.1)
Sunburn in the last 12 mo								
No	1662 (60.6)	498 968 (65.2)	1409 (51.1)	458 334 (57.9)	680 (48.6)	348 541 (54.6)	3751 (54.4)	1 305 842 (59.5)
Yes	1081 (39.4)	265 773 (34.8)	1348 (48.9)	333 793 (42.1)	719 (51.4)	289 751 (45.4)	3148 (45.6)	889 317 (40.5)
Tanning bed or booth in the last 12 mo								
No	2729 (99.7)	757 839 (99.8)	2739 (99.3)	787 723 (99.4)	1314 (93.9)	608 296 (95.3)	6782 (98.4)	2 153 859 (98.3)
Yes	8 (0.3)	1841 (0.2)	18 (0.7)	4404 (0.6)	85 (6.1)	29 995 (4.7)	111 (1.6)	36 240 (1.7)
Uses sunscreen on body								
Sometimes, rarely, or never	1226 (49.4)	350 307 (51.3)	1618 (64.3)	468 385 (65.4)	854 (68.9)	387 773 (69.2)	3698 (59.3)	1 206 466 (61.5)
Always or often	1258 (50.6	332 976 (48.7)	899 (35.7)	247 930 (34.6)	386 (31.1)	172 930 (30.8)	2543 (40.7)	753 836 (38.5)
Uses sunscreen on face								
Sometimes, rarely, or never	1215 (48.7)	342 182 (49.7)	1545 (61.3)	451 883 (63.0)	811 (65.4)	367 345 (65.5)	3571 (57.1)	1 161 410 (59.1)
Always or often	1280 (51.3)	345 998 (50.3)	977 (38.7)	265 160 (37.0)	429 (34.6)	193 358 (34.5)	2686 (42.9)	804 516 (40.9)
Wears hat								
Sometimes, rarely, or never	1727 (69.2)	473 565 (68.7)	1856 (73.6)	532 949 (74.7)	920 (74.3)	422 993 (75.6)	4503 (72.0)	1 429 508 (72.8)
Always or often	769 (30.8)	215 441 (31.3)	665 (26.4)	180 927 (25.3)	318 (25.7)	136 586 (24.4)	1752 (28.0)	532 954 (27.2)
Wears long pants or skirt								
Sometimes, rarely, or never	1946 (78.0)	529 527 (76.8)	1903 (75.5)	543 879 (75.9)	897 (72.5)	400 514 (71.6)	4746 (75.9)	1 473 920 (75.0)
Always or often	548 (22.0)	159 516 (23.2)	618 (24.5)	173 132 (24.1)	341 (27.5)	158 579 (28.4)	1507 (24.1)	491 228 (25.0)
Wears sunglasses								
Sometimes, rarely, or never	1743 (69.8)	489 356 (71.0)	1574 (62.4)	470 717 (65.7)	622 (50.2)	288 122 (51.4)	3939 (63.0)	1 248 195 (63.5)
Always or often	754 (30.2)	199 914 (29.0)	947 (37.6)	246 128 (34.3)	617 (49.8)	272 250 (48.6)	2318 (37.0)	718 292 (36.5)
Seeks shade								
Sometimes, rarely, or never	1586 (63.6)	421 009 (61.1)	1615 (64.1)	439 437 (61.3)	808 (65.3)	366 863 (65.6)	4009 (64.1)	1 227 309 (62.5)
Always or often	908 (36.4)	267 620 (38.9)	904 (35.9)	277 199 (38.7)	430 (34.7)	192 056 (34.4)	2242 (35.9)	736 875 (37.5)

^a^ In the Canadian Community Health Survey, the visible minority population consists mainly of the following groups: Arab, Black, Chinese, Filipino, Japanese, Korean, Latin American, South Asian, Southeast Asian, and West Asian.

Compared with older teens, adolescents aged 12 to 14 years were more likely to use sunscreen on the body and face and to wear hats (Figure). Female youths more frequently used sunscreen and sunglasses but also had paradoxically higher odds of sunburn and greater tanning bed use. Sunscreen use was associated with increased sunburn risk, particularly regular facial sunscreen use among White adolescents, while hat and long-clothing use were consistently protective. Youths from households earning at least $80 000 annually were more likely to use sunscreen and sunglasses but also had higher odds of sunburn and were less likely to wear long clothing. A race-income interaction (*P* = .04) revealed that among Indigenous adolescents, higher income was associated with greater odds of sunburn (OR, 2.530; 95% CI, 1.499-4.346).

**Figure.**
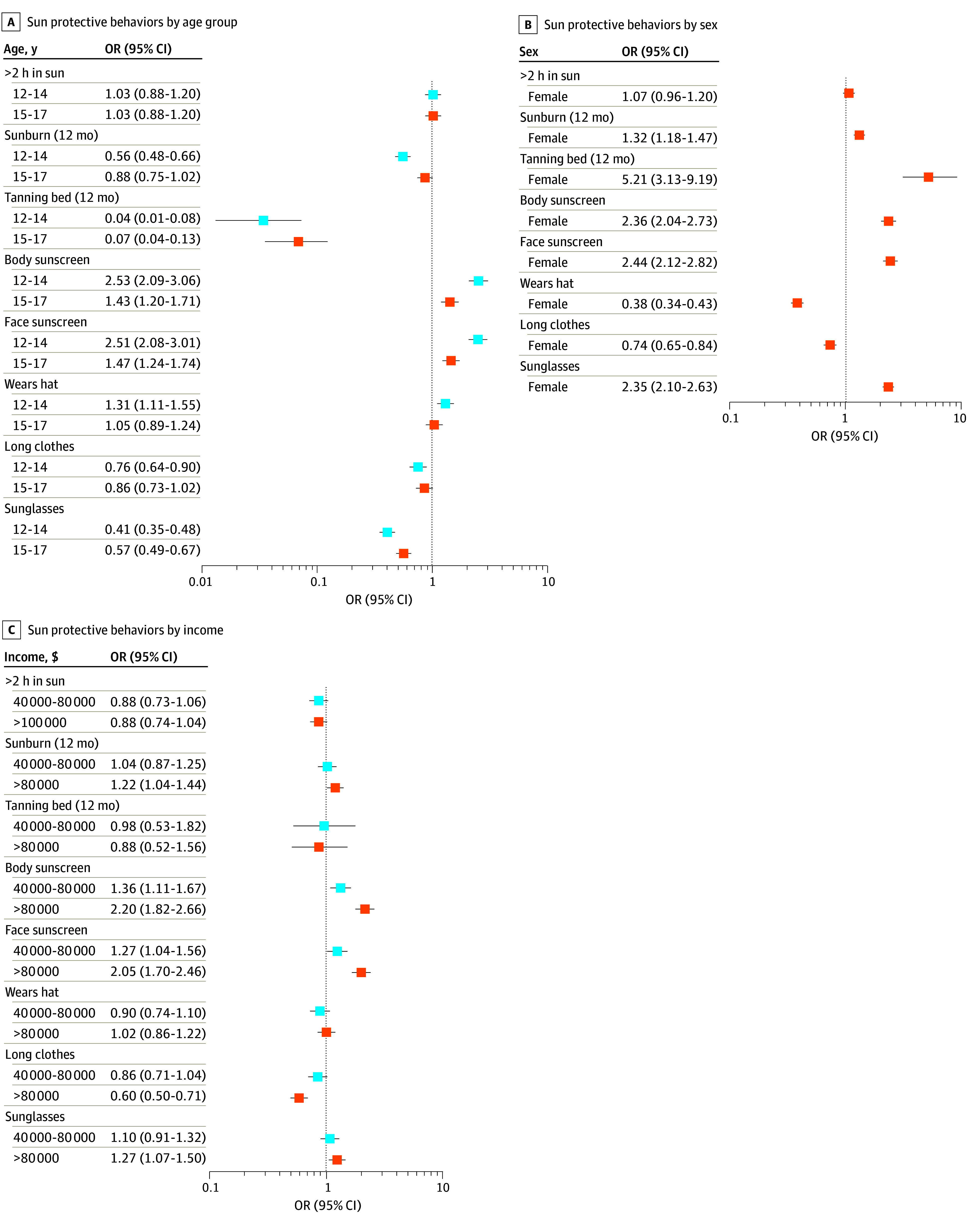
Sun Exposure and Sun Protective Behaviors by Age, Sex, and Income Forest plots compare sun exposure and protective behavior outcome variables by age group (reference, 18-19 years) (A), sex (reference, male) (B), and income (reference, $40 000) (C). Adjusted odds ratios (ORs) controlling for age, sex and race, where applicable, are presented.

## Discussion

This survey study found that Canadian adolescents have high sunburn prevalence and inconsistent sun protection, with elevated risk among older, female, higher-income, and Indigenous youths. The current 40.5% sunburn rate cannot be compared with previous data^3^ (45% in 1996 and 16% in 2006) due to differing samples. However, our results suggest little progress, or possible regression, in sun safety over 2 decades.

Compared with Canadian adults,^4^ adolescents remain more vulnerable to unsafe UV exposure. Our findings highlight a critical transition phase: children aged 12 to 14 years (under parental supervision) exhibit better sun protection, whereas behavior deteriorates after age 15 years, coinciding with autonomy and, by 18 to 19 years, increased artificial tanning. Similar patterns have been observed among adolescents in the US and Europe.^5^

Despite more frequent sunscreen use, female and higher-income youths experience more sunburn, illustrating the sunscreen paradox,^6^ where reliance on sunscreen may encourage prolonged UV exposure without adequate application or reapplication. Limitations include the cross-sectional design and self-reported data, which may introduce recall or social desirability bias. Some groups including remote, institutionalized, or unhoused youth are underrepresented in CCHS, limiting generalizability.

Canada has made limited progress in adolescent sun safety. Findings underscore the need to strengthen public health messaging beyond sunscreen-only approaches and to target the vulnerable transition into late adolescence, reinforcing comprehensive sun-safe behaviors.

## Notes

Supplement 1. eMethods eTable. Overall Missingness for Variables of Interest (Unweighted) eReferences

Supplement 2. Data Sharing Statement
