# Supplementary material for: Sun Exposure and Protection Practices Among Youths in Canada
Source: JAMA Netw Open. 2026 Jan 5;9(1):e2551872. doi: 10.1001/jamanetworkopen.2025.51872 (PMC12771215; doi:10.1001/jamanetworkopen.2025.51872)
Supplement: Supplement 1. — eMethods eTable. Overall Missingness for Variables of Interest (Unweighted) eReferences [file jamanetwopen-e2551872-s001.pdf]

Supplemental Online Content

Moustaqim-Barrette A, Elhaj H, Kanou M, Netchiporouk E, Litvinov IV. Sun exposure and protection practices among youth. JAMA Netw Open. 2026;9(1):e2551872. doi:10.1001/jamanetworkopen.2025.51872

eMethods.

eTable. Overall Missingness for Variables of Interest (Unweighted)

eReferences.

© 2025 Moustaqim-Barrette A et al. *JAMA Network Open*.

eMethods.

Survey design and population

This study utilized data from the 2015–2018 cycles of the Canadian Community Health Survey (CCHS)[1], administered annually by Statistics Canada. The CCHS is a nationally representative cross-sectional survey of Canadians aged ≥12 years that employs a multi-stage stratified cluster sampling strategy. Survey weights and 500 bootstrap replicate weights, provided by Statistics Canada, were applied in all analyses to ensure population representativeness and to account for the complex survey design. During 2015–2018, four provinces - Ontario, Quebec, Manitoba, and Saskatchewan - opted to include the sun safety module. The analytic cohort included respondents aged 12–19 years. For minors, informed consent was obtained from a parent or legal guardian, after which the survey was administered directly to the youth. Response rates for these cycles were ≥60% consistent with other national health surveys.

Variable definitions

Primary outcomes included: (1) time spent outdoors between 10 am and 4 pm on summer weekends (<2 vs ≥2 hours); (2) self-reported sunburn in the previous 12 months (yes/no); (3) tanning bed use in the past year (yes/no); (4) sunscreen use on face and body (always/often vs sometimes/rarely/never); (5) wearing a hat, long pants/skirts, or sunglasses outdoors (always/often vs sometimes/rarely/never). Exposures of interest included age (12–14, 15–17, 18–19 years), sex (biological sex at birth), self-identified race/ethnicity (White, Indigenous, or visible minority, defined per the Canadian Employment Equity Act), and total household income (<$40,000; $40–80,000; ≥$80,000).

Statistical analysis

Weighted frequency distributions were generated to describe sample characteristics. Complete case multivariable logistic regression models were used to examine associations between demographic variables and each outcome, adjusting for age, sex, and race/ethnicity. Missingness of data is described in Table S1. Predictors of sunburn in the past year were evaluated in a separate multivariable model including sun protective behaviours and demographic covariates. Variables were selected for inclusion based on bivariate associations ($p<0.25$) and/or conceptual relevance. Interaction terms of a priori interest (*e.g.,* income × race) were tested using likelihood ratio tests. Results are reported as adjusted odds ratios (ORs) with 95% confidence intervals (CIs). Analyses were conducted in R version 4.3.3[2].

eTable. Overall missingness for variables of interest (unweighted).			
**Variable**	**Missing (n)**	**Total (n)**	**Missing (%)**
Ever uses sunscreen on body	898	7,139	12.58
Wears long pants/skirts outdoors	886	7,139	12.41
Wears hat outdoors	884	7,139	12.38
Ever uses sunscreen on face	882	7,139	12.35
Wears sunglasses outdoors	882	7,139	12.35
Race (binary)	726	7,139	10.17
Spent >2h/day in sun	335	7,139	4.69
Used tanning bed in past 12 months	246	7,139	3.45
Had sunburn in past 12 months	240	7,139	3.36
Household income	45	7,139	0.63
Age group	0	7,139	0.00
Sex	0	7,139	0.00

eReferences

1. Government of Canada SC. Canadian Community Health Survey - Annual Component (CCHS). March 3, 2022. Accessed January 31, 2025. https://www23.statcan.gc.ca/imdb/p2SV.pl?Function=assembleDESurv&DECId=1323413&RepClass=591&Id=1383236&DFId=180541

2. R Core Team. R: A language and environment for statistical computing. Published online 2021. Accessed December 30, 2024. https://www.R-project.org/

© 2025 Moustaqim-Barrette A et al. *JAMA Network Open*.
