# Supplementary material for: Sun Exposure and Protection Practices Among Youths in Canada
Source: JAMA Netw Open. 2026 Jan 5;9(1):e2551872. doi: 10.1001/jamanetworkopen.2025.51872 (PMC12771215; doi:10.1001/jamanetworkopen.2025.51872)
Supplement: Supplement 2. — Data Sharing Statement [file jamanetwopen-e2551872-s002.pdf]

Data Sharing Statement

Moustaqim-Barrette. Sun Exposure and Protection Practices Among Youths in Canada. *JAMA Netw Open*. Published January 05, 2026. doi:10.1001/jamanetworkopen.2025.51872

Data
Data available: No

Additional Information
Explanation for why data not available: All original data is available from Statistics Canada.
